# Anti-*Mycobacterium tuberculosis* Activity of Esters of Quinoxaline 1,4-Di-*N*-Oxide

**DOI:** 10.3390/molecules23061453

**Published:** 2018-06-15

**Authors:** Isidro Palos, Julieta Luna-Herrera, Edgar E. Lara-Ramírez, Alejandra Loera-Piedra, Emanuel Fernández-Ramírez, Ma. Guadalupe Aguilera-Arreola, Alma D. Paz-González, Antonio Monge, Baojie Wan, Scott Franzblau, Gildardo Rivera

**Affiliations:** 1 Unidad Académica Multidisciplinaria Reynosa-Rodhe, Universidad Autónoma de Tamaulipas, Carr. Reynosa-San Fernando, s/n, Reynosa 88779, Mexico; isi_palos@hotmail.com; 2 Departamento de Inmunología, Escuela Nacional de Ciencias Biológicas, Instituto Politécnico Nacional, Ciudad de México 11340, Mexico; julietalunah@hotmail.com (J.L.-H.); ide_shadows@hotmail.com (A.L.-P.); g.u.ero69@hotmail.com (E.F.-R.); 3 Unidad de Investigación Biomédica de Zacatecas, Instituto Mexicano del Seguro Social (IMSS), Alameda Trinidad García de la Cadena, s/n, Zacatecas 98000, Mexico; elarar0700@hotmail.com; 4 Departamento de Microbiología, Escuela Nacional de Ciencias Biológicas, Instituto Politécnico Nacional, Ciudad de México 11340, Mexico; lupita_aguilera@hotmail.com; 5 Laboratorio de Biotecnología Farmacéutica, Centro de Biotecnología Genómica, Instituto Politécnico Nacional, Boulevard del Maestro, s/n, Esq. Elías Piña, Reynosa 88710, Mexico; apazg@ipn.mx; 6 Unidad de Investigación y Desarrollo de Medicamentos, Centro de Investigación en Farmacobiología Aplicada (CIFA), Universidad de Navarra, C/Irunlarrea s/n, 31080 Pamplona, Spain; amonge@unav.es; 7 Institute for Tuberculosis Research, College of Pharmacy, University of Illinois at Chicago, 833 S. Wood St., Chicago, IL 60612, USA; baojie@uic.edu (B.W.); sgf@uic.edu (S.F.)

**Keywords:** esters, quinoxaline 1,4-di-*N*-oxide, *Mycobacterium tuberculosis*, DNA gyrase, drug resistance

## Abstract

Tuberculosis continues to be a public health problem in the world, and drug resistance has been a major obstacle in its treatment. Quinoxaline 1,4-di-*N*-oxide has been proposed as a scaffold to design new drugs to combat this disease. To examine the efficacy of this compound, this study evaluates methyl, ethyl, isopropyl, and n-propyl esters of quinoxaline 1,4-di-*N*-oxide derivatives in vitro against *Mycobacterium tuberculosis* (pansusceptible and monoresistant strains). Additionally, the inhibitory effect of esters of quinoxaline 1,4-di-*N*-oxide on *M. tuberculosis* gyrase supercoiling was examined, and a stability analysis by ultra performance liquid chromatography-tandem mass spectrometry (UPLC-MS) was also carried out. Results showed that eight compounds (**T-007**, **T-018**, **T-011**, **T-069**, **T-070**, **T-072**, **T-085** and **T-088**) had an activity similar to that of the reference drug isoniazid (minimum inhibitory concentration (MIC) = 0.12 µg/mL) with an effect on nonreplicative cells and drug monoresistant strains. Structural activity relationship analysis showed that the steric effect of an ester group at 7-position is key to enhancing its biological effects. Additionally, **T-069** showed a high stability after 24 h in human plasma at 37 °C.

## 1. Introduction

According to the World Health Organization (WHO), tuberculosis (TB) continues to be one of the leading infectious diseases in the world [1]. First- and second-line drugs are available for the treatment of the disease. However, the present therapy has been ineffective due to its long duration, as well as the emergence of resistance to these drugs [2]. In view of the significance of TB as an infectious disease and the increasing incidence of resistant strains, the development of new drugs for the treatment of TB is urgently needed. 

Quinoxalines are a series of compounds with diverse biological activities that have what is considered a privileged structure [3]. Carta and collaborators proposed quinoxaline 1,4-di-*N*-oxide as a scaffold for the development of new antituberculosis drugs [4]. In particular, Monge’s research group reported a series of quinoxaline 1,4-di-*N*-oxide derivatives with excellent antituberculosis activity in resistant and multiresistant strains of *Mycobacterium tuberculosis* (*M. tuberculosis*) as well as nonreplicative cells in in vitro and in vivo models [5,6]. Interestingly, the change of the 2-carbonitrile by the 2-carboxylate group increased the solubility of the compounds and their biological activity [7]. Analysis of the structure-activity relationship (SAR) of quinoxaline 1,4-di-*N*-oxide derivatives clearly indicates that at 2- and 7-position on the quinoxaline ring, carboxylate and electro-attracting groups (halogen atoms), respectively, are preferred. Also, a methyl group at 3-position is a good option for antitubercular activity [8]. Additionally, quinoxaline 1,4-di-*N*-oxide derivatives have an effect against nonreplicating persistent (NRP) bacteria [9]. A study by Pan et al. showed that quinoxaline 1,4-di-*N*-oxide derivatives have a potent antitubercular activity (minimum inhibitory concentration (MIC) < 0.4 μg/mL) and null cytotoxicity on Vero cell lines [10]. However, in general, quinoxaline 1,4-di-*N*-oxide derivatives show solubility problems. In line with the development of new, more effective, and less toxic antituberculosis agents, our research group in this work propose the incorporation of an ester group (methyl, ethyl, isopropyl, and n-propyl) at 7-position on the quinoxaline 1,4-di-*N*-oxide ring as a strategy to enhance the solubility and biological activity of these derivatives.

Additionally, it has been reported that quinoxaline 1,4-di-*N*-oxide derivatives have a novel mechanism of action unrelated to current antitubercular drugs [11]. A proposed mechanism of action indicates that antimicrobial quinoxalines prevent the synthesis of RNA by binding to the CpG site on DNA [12]. However, a study examining compounds derived from 2,3-dichloroquinoxaline has indicated that they may be inhibitors of the enzyme chorismate mutase [13]. In addition to the mechanisms of action described, Radwan et al. have determined that quinoxaline 1,4-dioxide derivatives are capable of interacting on the active site of DNA gyrase of *M. tuberculosis* [14]. Therefore, in this study, a inhibitory analysis of esters of quinoxaline 1,4-di-*N*-oxide derivatives on *M. tuberculosis* gyrase supercoiling was done to understand their potential mechanism of action. Finally, a chromatographic analysis to test plasma stability of esters of quinoxaline 1,4-d-*N*-oxide was also carried out. 

## 2. Results and Discussion

### 2.1. Biological Activity

A common procedure to obtain quinoxaline 1,4-di-*N*-oxide derivatives is the use of benzofuroxane *N*-oxide as a principal reagent [15]. Additionally, benzofuroxane *N*-oxide is a heterocyclic compound with antitubercular activity [16]. Therefore, as a first step, benzofuroxane-*N*-oxide derivatives were used to obtain esters of quinoxaline 1,4-di-*N*-oxide, which were also evaluated in vitro on *M. tuberculosis* strain H37Rv and the NRP strain. Results are shown in Table 1. SAR analysis showed that the compound **T-046** with a hydrogen atom at R_1_ position on the benzofuroxane *N*-oxide ring had a lower MIC value (58.5 µg/mL). However, when a carboxylate group was added at R_1_-position, the biological activity was enhanced sevenfold (compound **T-014**, MIC = 7.6 µg/mL). This effect increased when a carboxylate group with an aliphatic linear substituent was incorporated at the same position on the benzofuroxane *N*-oxide ring (**T-074**, MIC = 0.87 µg/mL). These results confirm that the steric effect at R_1_-position modulates anti-*M. tuberculosis* activity. Additionally, a second substitution at R_2_-position drastically reduced the activity (**T-036**, MIC >100 µg/mL). These results show that the benzofuroxane-5-carboxylate *N*-oxide ring is a good scaffold to develop new antitubercular agents.

Following on our main objective (esters of quinoxaline 1-4-di-*N*-oxide derivatives), 18 compounds of methyl and ethyl ester derivatives were evaluated on *M. tuberculosis* strain H37Rv. Results are shown in Table 2. All compounds of the methyl ester series showed good anti-*M. tuberculosis* activity (MIC < 0.35 µg/mL). SAR analysis of the methyl series, showed that compounds with a carboxylate (methyl and ethyl) group at R_1_-position and a methyl group at R_2_-position had high activity (MIC < 0.5 µg/mL). However, acetyl, benzoyl, and arylcarboxamide groups at R_1_-position decreased the effect. On the other hand, quinoxaline 1,4-di-*N*-oxide derivatives with a trifluoromethyl group at R_2_-position also showed good anti-*M. tuberculosis* activity (MIC < 2.0 µg/mL) in spite of the presence of a carbonyl group with aliphatic or aromatic substitutes at R_1_-position. A comparison between analog compounds (**T-003** and **T-018**) with a methyl and trifluoromethyl group at R_2_-position, respectively, showed that the electronegative properties of trifluoromethyl enhance their biological activity. Compound **T-018** showed the best biological activity (MIC = 0.15 µg/mL), a value similar to the reference drug isoniazid (MIC = 0.12 µg/mL). Also, this compound showed the best activity (low oxygen recovery assay (LORA) MIC = 0.34 µg/mL) against the NRP *M. tuberculosis* strain. Analyzing all LORA results of quinoxaline 1,4-di-*N*-oxide derivatives, these compounds had the same biological behavior on *M. tuberculosis* strain H37Rv; therefore, substitutions at R_1_- and R_2_-position also affect biological activity on the NRP strain.

In the ethyl ester series in general, all quinoxaline 1,4-di-*N*-oxide derivatives showed good anti-*M. tuberculosis* activity (MIC < 2.5 µg/mL) except compounds **T-026** and **T-045,** which had ten times less activity (MIC > 22 µg/mL). These compounds have an aliphatic linear substitution or a free amino group at R_1_-position with a trifluoromethyl and methyl group at R_2_-position, respectively. Compound **T-015** with a carboxyethyl and methyl group at R_1_- and R_2_-position, respectively, showed a MIC of 0.50 µg/mL. In addition, compounds with a trifluoromethyl group at R_2_-position showed better anti-*M. tuberculosis* activity with an acetyl or aromatic group at R_1_-position. Compounds **T-007** and **T-011** showed MIC values (0.14 and 0.10 µg/mL) similar to that of isoniazid (MIC = 0.12 µg/mL). These compounds have a thiophene and a naphthyl group, respectively. These groups could consider a bioisostere from a phenyl group present in the best compound (**T-018**) of the methyl ester series. Additionally, **T-007**, **T-011,** and **T-018** showed the best effect on the NRP *M. tuberculosis* strain. An analysis between the methyl and ethyl series showed that a substitution at R_3_-position does not affect biological activity; for example, compounds **T-008** and **T-016** showed a similar MIC value. Additionally, the ethyl quinoxaline 1,4-di-*N*-oxide series confirm that substitutions at R_1_- and R_2_-position also affect the biological behavior on the NRP *M. tuberculosis* strain.

**Figure 1:**
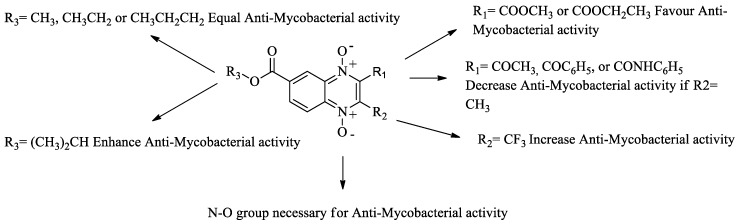
A structure-activity relationship analysis from esters of quinoxaline-7-carboxylate 1,4-di-*N*-oxide as antimycobacterial agents.

Results shown in Table 2 confirm that methyl and ethyl ester groups at R_3_-position on the quinoxaline 1,4-di-*N*-oxide ring do not produce changes in anti-*M. tuberculosis* activity. However, these results (at R_1_- and R_2_-position) also suggest that steric effects modify biological activity. Therefore, we proposed obtaining two new series with an aliphatic substituent (isopropyl and n-propyl) at R_3_-position to confirm a positive, negative, or null effect on biological activity (Table 3). SAR analysis of the isopropyl series confirmed that a carboxylate (methyl and ethyl) group at R_1_-position enhanced anti-*M. tuberculosis* activity. As in the previous methyl and ethyl series, benzoyl, arylcarboxamide, or amine groups at R_1_-position reduced the activity with a methyl group at R_2_-position on the quinoxaline 1,4-di-*N*-oxide ring. Interestingly, an isopropyl group at R_1_-position drastically decreased biological activity. This confirms that steric effects are important in anti-*M. tuberculosis* activity. Also, in the isopropyl series, compounds with a trifluoromethyl group at R_2_-position showed the best activity. In particular, compound **T-069** showed the best MIC (0.08 µg/mL) of all the quinoxaline 1,4-di-*N*-oxide derivatives. This compound is an analogue of compound **T-011** (ethyl series), although compound **T-069** had a MIC value ten times higher. Therefore, the isopropyl group at R_3_-position is a key factor to enhance anti-*M. tuberculosis* activity. In the n-propyl series, the SAR partner is the same as the previous series (methyl, ethyl and isopropyl). The best compound in this series was **T-089** (an analogue of **T-011** and **T-069**) with a MIC of 0.12 µg/mL on strain H37Rv and 0.15 µg/mL on NRP *M. tuberculosis* strain. A SAR analysis of all esters of quinoxaline 1,4-di-*N*-oxide is shown in Figure 1.

Finally, eight compounds (MIC < 0.30 µg/mL) from all esters of quinoxaline 1,4-di-*N*-oxide derivatives were selected to be tested against monoresistant *M. tuberculosis* and some nontuberculous Mycobacterium (NTM) strains. Additionally, the half maximal cytotoxicity concentration (CC_50_) on mammalian cell macrophages and the selectivity index (SI) were determined. The results are shown in Table 4. In general, all compounds had similar MICs values on *M. tuberculosis* and monoresistant strains. This suggests that the mechanism of action of esters of quinoxaline 1,4-di-*N*-oxide derivatives is different from reference drugs. Although our compounds had good antimycobacterial activity, none of the quinoxaline 1,4-di-*N*-oxide derivatives showed better MIC values than rifampicin in monoresistant strains, except against *M. tuberculosis* strain H37Rv RR. Compounds **T-022** and **T-088** showed the best biological activity on this strain and the best SI value (SI > 70). Activity against the NTM strains was not as good as with the tuberculosis strains; only compound **T-085** was active against all NTM strains, and the slow grower NTM *M. avium* strain was susceptible to all compounds except **T-088**. These results confirm that these esters of quinoxaline 1,4-di-*N*-oxide can be considered for development of new pharmacological options for the treatment of TB susceptible or drug resistant.

### 2.2. Inhibitory Assay

DNA gyrases are enzymes within the class of type II topoisomerase, which form a heterotetramer composed of four subunits encoded by the *gyrA* and *gyrB* gene [17]. This enzyme catalyzes the unwinding of double-stranded DNA through the introduction of negative supercoils. This process is blocked by fluoroquinolones causing the formation of covalent enzyme-DNA adducts, which leads to chromosome fragmentation and cell death in bacteria. Thus, DNA gyrases are known targets for fluoroquinolones. However, some studies have hypothesized that quinoxaline derivatives could inhibit DNA gyrases from *M. tuberculosis* [14,18]. Keeping in pace with these conjectures, we analyzed the inhibitory effect of esters of quinoxaline 1,4-di-*N*-oxide derivatives on *M. tuberculosis* gyrase supercoiling. The compounds tested were: **T-003**, **T-013**, **T-018,** and **T-038** from the methyl series; **T-011** and **T-012** from ethyl series; **T064**, **T-069,** and **T-108** from isopropyl series; and **T-114** and **T-125** from n-propyl series. However, none of the compounds showed an inhibitory effect on *M. tuberculosis* gyrase supercoiling at 50 μM. Moxifloxacin, the positive control, showed an IC_50_ value of 9.44 μM. The compounds **T-011**, **T-018**, **T-038**, **T-069,** and **T-114** showed the trifluoromethyl group at R_2_-position, with this not interfering with the union of DNA-protein. This was observed in earlier studies employing carcinogenic polycyclic aromatic compounds with trifluoromethyl group [19]. Based on these results, esters of quinoxaline 1,4-di-*N*-oxide derivatives may not be *M. tuberculosis* gyrase supercoiling inhibitors, suggesting that these esters of quinoxaline 1,4-di-*N*-oxide derivatives have another mechanism of action that needs to be explored.

### 2.3. Stability Analysis by UPLC-MS

**Figure 2:**
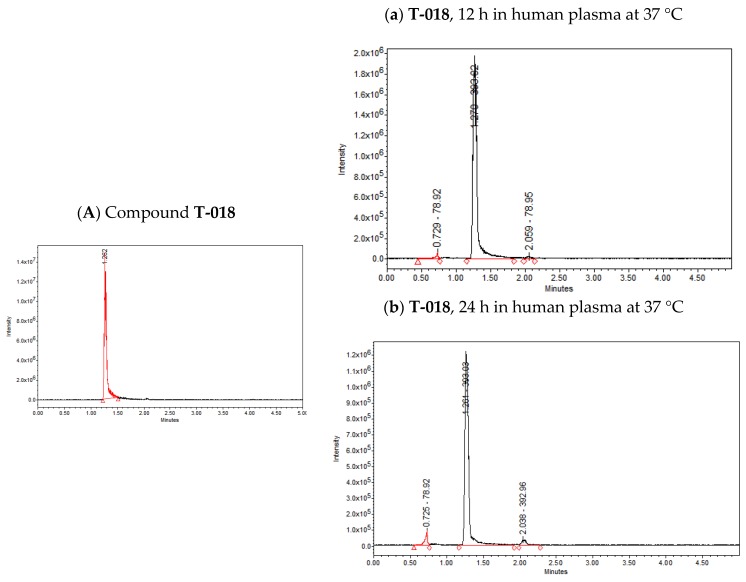

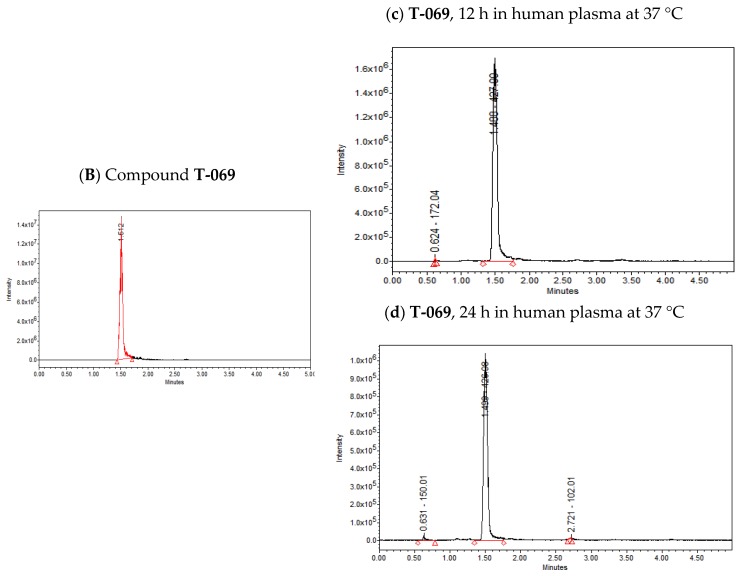
Ultra performance liquid chromatography (UPLC) of (**A**) compound **T-018** and (**B**) compound **T-069**. (**a**) **T-018** after 12 h in human plasma at 37 °C; (**b**) **T-018** after 24 h in human plasma °C; (**c**) **T-069** after 12 h in human plasma at 37 °C; and (**d**) **T-069** after 24 h in human plasma at 37 °C.

The presence of ester groups at 7-position on the quinoxaline 1,4-di-*N*-oxide ring could suggest that these compounds will not be stable when tested in animal models. Therefore, a simple assay to test the stability of compounds **T-018** and **T-069**—methyl and isopropyl ester derivatives, respectively—was performed using ultra performance liquid chromatography-tandem mass spectrometry (UPLC-MS). The results are shown in Figure 2. Figure 2A, shows only one peak with a time retention of 1.26 min corresponding to compound **T-018** (*m*/*z* = 393.02). After 12 h in human plasma, two new low intensity peaks are seen (time retention= 0.72 and 2.05 min) (Figure 2a). These peaks, which increased in intensity after 24 h (Figure 2b) represented 4.82 and 4.12%, respectively, suggesting a low degradation of compound **T-018**. In Figure 2B, only one peak was observed with a time retention of 1.51 min corresponding to compound **T-069** (*m*/*z* = 427.02). After 12 h, a second peak is seen at 0.62 min (Figure 2c). Finally, after 24 h, another peak occurs at 2.72 min (Figure 2d). Both peaks at 24 h represent 0.83% and 2.20%, respectively, suggesting that **T-069** had a lower degradation. These results show that esters of quinoxaline 1,4-di-*N*-oxide are stable in human plasma after 24 h.

## 3. Materials and Methods

### 3.1. Chemical Synthesis

All compounds from the methyl, ethyl, isopropyl, and n-propyl ester series of quinoxaline 1,4-*N*-oxide were obtained following the procedure previously reported by Gomez-Caro et al. [20]. These compounds were analyzed by IR, ^1^H-NMR and elemental analysis and have been previously reported [21,22,23,24].

### 3.2. Antitubercular Assays

The antitubercular activity (MIC values) was assessed in vitro against *Mycobacterium tuberculosis* strain H37Rv ATCC 27294 according to a modified microplate Alamar blue assay (MABA) [25]. The assays were performed in triplicate independent experiments. The standard *M. tuberculosis* strain H37Rv was tested with known reference drugs rifampicin and isoniazid. The lowest drug concentration effecting an inhibition of 90% was considered as MIC. Additionally, an in vitro LORA test was done following the procedure previously reported by Cho et al. [26]. Antituberculosis activity testing against drug monoresistant strains (ATCC35822, ATCC35838, ATCC35837, and ATCC35820) or NTM clinical isolates was performed as described earlier by Luna-Herrera et al. [27].

### 3.3. Selectivity Index

The determination of the half maximal cytotoxicity concentration (CC_50_) of the compounds **T-006**, **T-011**, **T-018**, **T-022**, **T-069**, **T-085**, **T-088,** and **T-089** was carried out on the mouse macrophage cell line J774A.1 (ATCC TIB-67). Briefly, cell monolayers were prepared in 96-well plates with 10,000 cells per well in Ham’s F-12 medium supplemented with 10% Fetal bovine serum (FBS, By products, Guadalajara, Mexico) and antibiotics (penicillin and gentamicin). Five concentrations from 100 to 0.5 μg/mL of each of the compounds were tested. The cells were incubated for a period of 6 h (^−^) or 96 h (^+^). Before completing this period, 20 μL of Alamar blue solution was added to each well, quantifying the relative fluorescence units in a fluorometer (Fluoroskan Ascent FL, Labsystems). The percentage of cytotoxicity at each concentration was determined by comparing the values against the control of cells without treatment. The CC_50_ was determined with the Probit regression analysis (MedCalc Statistical Software version 18.5, Ostend, Belgium). Selectivity index was calculated as the ratio of the CC_50_ on the macrophage cell line J774A.1 and the MIC value against *M. tuberculosis* strain H37Rv [28].

### 3.4. Enzymatic Assay

#### 3.4.1. Assay Set Up

The activity of the enzyme was determined prior to the testing of the quinoxaline 1,4-di-*N*-oxide derivatives and 1 U was defined as the amount of enzyme required to just fully supercoil the substrate under test conditions. Compounds were tested at 50 μM. Final DMSO concentration in the assays was 1% (*v*/*v*).

#### 3.4.2. *M. tuberculosis* Gyrase Supercoiling

1 U of *M. tuberculosis* gyrase (GyrA and Gyr B subunits) (final concentration in assay 50 nM) was incubated with 0.5 μg of relaxed pBR322 DNA in a 30 μL reaction at 37 °C for 30 min under the following conditions: 50 mM HEPES. KOH (pH 7.9), 6 mM magnesium acetate, 4 mM DTT, 1 mM ATP, 100 mM potassium glutamate, 2 mM spermidine and 0.05 mg/mL albumin.

Each reaction was stopped by the addition of 30 μL chloroform/iso-amyl alcohol (26:1) and 20 μL Stop Dye (40% sucrose, 100 mM Tris.HCl (pH 7.5), 10 mM EDTA, 0.5 μg/mL bromophenol blue), before being loaded on a 1.0% TAE (Tris.acetate 0.04 mM, EDTA 0.002 mM) gel run at 80 V for 2 h.

#### 3.4.3. Data Acquisition and Analysis

Bands were visualized by ethidium staining for 10 min, destained for 10 min in water, analyzed by gel documentation equipment (Syngene, Cambridge, UK), and quantified using Syngene software (Genetools version 4.00 (1997–1998), Syngene, A division of Synoptics Corp, Cambridge, UK).

Raw gel data (fluorescent band volumes) collected from Syngene’s GeneTools gel analysis software were converted to a % of the 100% control (the supercoiled enzyme plus DMSO control DNA band). These were analyzed using SigmaPlot Version 13.0 (Systat Software Inc., London, UK; 2016).

### 3.5. Chromatographic Analysis

Compounds **T-018** and **T-069** were selected to analyze their stability in human plasma. A total of 1 mg of each of the two compounds was dissolved in 1 mL of dichlorometane. Then, 0.1 mL was added to 0.9 mL of 0.1% formic acid in acetonitrile for analysis by UPLC with an ACQUITY QDa mass detector from Waters (Milford, MA, USA) under the following conditions: column: ACQUITY UPLC^®^ BEH C_18_ 1.7 µm, 2.1 × 100 mm; mobile phase: 0.1% formic acid/acetonitrile; run time: 5 min; flow rate: 0.3 mL/min; injection volume: 0.5 µL; temperature: 40 °C. After that, 3 mL of human plasma was obtained from a volunteer under medical supervision and following the standard procedures. Then, 1 mg of **T-018** and **T-069** were dissolved with DMSO 2% and added to 1 mL of human plasma. Samples were incubated at 37 °C for 12 and 24 h. **T-018** and **T-069** were obtained by liquid extraction with dichloromethane (10 mL) three times and the solvent was eliminated for vacuum pressure. After that, 1 mL of dichlorometane was added and 0.1 mL was added to 0.9 mL of 0.1% formic acid in acetonitrile and analyzed by UPLC-MS. Percentage of degradation was calculated considering the area under the curve of each peak/the area under the curve of all peaks × 100% at 24 h only.

## 4. Conclusions

In this study, our results confirmed that esters of quinoxaline 1,4-di-*N*-oxide have good anti-*M. tuberculosis* activity (MIC < 5 μg/mL) except for five compounds (**T-045**, ethyl series; **T-066** and **T-108**, isopropyl series; **T-091** and **T-0125**, n-propyl series). Additionally, the best compounds (**T-007**, **T-011**, **T-018**, **T-069**, **T-070**, **T-072**, **T-085,** and **T-088**) with a MIC value of ≤0.15 μg/mL showed biological activity on both the nonreplicative cells and *M. tuberculosis* monoresistant strains. Structure-activity analysis showed that the compounds with better biological activity were obtained when carboxylate, trifluoromethyl, and isopropyl groups were present at R_1_, R_2_ and R_3_-position, respectively, on the quinoxaline 1,4-di-*N*-oxide ring. An enzymatic assay showed that these compounds cannot inhibit *M. tuberculosis* gyrase supercoiling. Also, these compounds showed a low degradation in human plasma. Therefore, esters of quinoxaline 1,4-di-*N*-oxide are a good option for developing new antitubercular agents.

## Figures and Tables

**Table 1:**
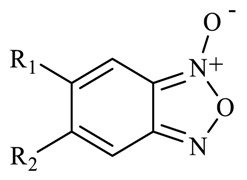
Biological activity of benzofuroxane *N*-oxide derivatives using the microplate Alamar blue assay (MABA) against *Mycobacterium tuberculosis* strain H37Rv and the low oxygen recovery assay (LORA) against the nonreplicating persistent strain.

Code	R_1_	R_2_	MABA MIC (µg/mL)	LORA MIC (µg/mL)
**T-014**	CH_3_OOC	H	7.6	12.98
**T-036**	CH_3_OOC	CH_3_O	>128	ND
**T-046**	H	H	58.5	46.97
**T-063**	(CH_3_)_2_CHOOC	H	1.4	3.04
**T-074**	CH_3_CH_2_CH_2_OOC	H	0.87	4.62
RMP			0.03	0.89
INH			0.12	>128

ND = not determined; RMP: rifampicin; INH: isoniazid.

**Table 2:**
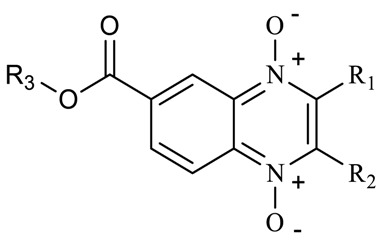
Biological activity of methyl and ethyl quinoxaline-7-carboxylate 1,4-di-*N*-oxide derivatives using the MABA against *Mycobacterium tuberculosis* strain H37Rv and the LORA against the nonreplicating persistent strain.

Code	R_1_	R_2_	R_3_	MABA MIC (µg/mL)	LORA MIC (µg/mL)
**T-003**	COC_6_H_5_	CH_3_	CH_3_	3.47	1.49
**T-004**	CO-phenyl	CH_3_	CH_3_CH_2_	2.32	0.61
**T-006**	COCH_3_	CF_3_	CH_3_CH_2_	0.29	0.42
**T-007**	CO-napthyl	CF_3_	CH_3_CH_2_	0.14	0.43
**T-011**	CO-thienyl	CF_3_	CH_3_CH_2_	0.10	0.21
**T-012**	CONHC_6_H_5_	CH_3_	CH_3_	1.07	0.86
**T-013**	COOCH_2_CH_3_	CH_3_	CH_3_	0.47	0.54
**T-015**	COOCH_2_CH_3_	CH_3_	CH_3_CH_2_	0.50	0.49
**T-018**	CO-phenyl	CF_3_	CH_3_	0.15	0.34
**T-022**	COOCH_3_	CH_3_	CH_3_	0.29	0.56
**T-026**	COCH_2_CH_3_	CF_3_	CH_3_CH_2_	22.5	2.5
**T-034**	COCH_3_	CH_3_	CH_3_	1.1	0.7
**T-037**	COOCH_2_CH_3_	CH_2_COOCH_2_CH_3_	CH_3_	1.2	1.9
**T-038**	COCH_2_CH_3_	CF_3_	CH_3_	<0.4	ND
**T-039**	COCH(CH_3_)_2_	CF_3_	CH_3_	1.54	0.83
**T-042**	COOCH_2_CH_3_	C_6_H_5_	CH_3_	1.0	0.5
**T-043**	COOCH_2_CH_3_	C_6_H_5_	CH_3_CH_2_	0.54	0.47
**T-045**	CONH_2_	CH_3_	CH_3_CH_2_	29.83	100.29
RMP				0.03	0.89
INH				0.12	>128

ND = not determined; RMP: rifampicin; INH: isoniazid.

**Table 3:**
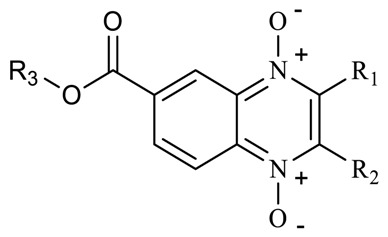
Biological activity of isopropyl and n-propyl quinoxaline-7-carboxylate 1,4-di-*N*-oxide derivatives using the MABA against *Mycobacterium tuberculosis* strain H37Rv and the LORA against nonreplicating persistent strain.

Code	R_1_	R_2_	R_3_	MABA MIC (µg/mL)	LORA MIC (µg/mL)
**T-064**	COOCH_3_	CH_3_	(CH_3_)_2_CH	0.58	0.56
**T-065**	COOCH_2_CH_3_	CH_3_	(CH_3_)_2_CH	0.7	0.5
**T-066**	COOC(CH_3_)_3_	CH_3_	(CH_3_)_2_CH	68.6	>100 (77%)
**T-067**	COOCH_2_CH_3_	CH_2_COOCH_2_CH_3_	(CH_3_)_2_CH	3.41	2.47
**T-069**	CO-thienyl	CF_3_	(CH_3_)_2_CH	0.08	0.23
**T-070**	COCH_3_	CF_3_	(CH_3_)_2_CH	0.14	0.24
**T-071**	CO-phenyl	CF_3_	(CH_3_)_2_CH	1.19	0.64
**T-072**	CO-napthyl	CF_3_	(CH_3_)_2_CH	0.15	0.51
**T-073**	CO-furyl	CF_3_	(CH_3_)_2_CH	0.7	0.6
**T-084**	COCH_3_	CH_3_	(CH_3_)_2_CH	0.8	0.6
**T-085**	COCH(CH_3_)_2_	CF_3_	(CH_3_)_2_CH	0.13	0.13
**T-088**	COOCH_3_	CH_3_	CH_3_CH_2_CH_2_	0.14	0.27
**T-089**	CO-thienyl	CF_3_	CH_3_CH_2_CH_2_	0.12	0.15
**T-090**	COOCH_2_CH_3_	CH_3_	CH_3_CH_2_CH_2_	0.8	ND
**T-091**	COC(CH_3_)_3_	CH_3_	CH_3_CH_2_CH_2_	>100 (33%)	ND
**T-097**	CONH-phenyl	CH_3_	(CH_3_)_2_CH	>20 (6%)	>20 (33%)
**T-098**	CO-phenyl	CH_3_	(CH_3_)_2_CH	4.59	2.87
**T-107**	COC(CH_3_)_3_	C(CH_3_)_3_	(CH_3_)_2_CH	2.1	ND
**T-108**	CONH_2_	CH_3_	(CH_3_)_2_CH	22.5	ND
**T-111**	COCH(CH_3_)_2_	CF_3_	CH_3_CH_2_CH_2_	1.5	ND
**T-112**	COOCH_2_CH_3_	COCOOCH_2_CH_3_	CH_3_CH_2_CH_2_	1.4	ND
**T-113**	COCH_3_	CF_3_	CH_3_CH_2_CH_2_	3.0	ND
**T-114**	CO-furyl	CF_3_	CH_3_CH_2_CH_2_	2.9	ND
**T-115**	CO-phenyl	CF_3_	CH_3_CH_2_CH_2_	1.5	ND
**T-124**	COOCH_2_CH_3_	CF_3_	CH_3_CH_2_CH_2_	3.0	ND
**T-125**	CONH-phenyl	CH_3_	CH_3_CH_2_CH_2_	12.3	ND
**T-126**	COCH_3_	CH_3_	CH_3_CH_2_CH_2_	3.0	ND
**T-130**	CONH_2_	CH_3_	CH_3_CH_2_CH_2_	2.9	ND
**T-132**	CO-phenyl	CH_3_	CH_3_CH_2_CH_2_	2.3	ND
RMP				0.03	0.89
INH				0.12	>128

ND = not determined; RMP: rifampicin; INH: isoniazid.

**Table 4:** Minimum inhibitory concentration (MIC in μg/mL) of esters of quinoxaline 1,4-di-*N*-oxide derivatives on monoresistant *M. tuberculosis* and some nontuberculous Mycobacterium strains.

Code	*M. tb* H37Rv	*M. tb* H37Rv IR	*M. tb* H37Rv ER	*M. tb* H37Rv SR	*M. tb* H37Rv RR	*M. fortuitum*	*M. abscessus*	*M. chelonae*	*M. avium*	*M. smegmatis*	CC_50_	SI
**T-006 ~**	2.0	1.25	1.25	5	1.25	>10	>10	>10	2.5	>10	>100 ^+^	>50
**T-011 ~**	0.5	<0.31	<0.31	0.625	<0.31	2.5	>10	>10	<0.31	0.62	1.67 ^−^	3.34
T-018 *	1.0	0.62	0.62	1.25	0.625	>10	5	>10	2.5	2.5	35.37 ^−^	35.37
T-022 *	0.5	0.62	0.62	0.625	<0.31	2.5	>10	2.5	2.5	0.62	86.25 ^+^	172.5
**T-069 ¤**	1.0	0.62	1.25	1.25	1.25	>10	>10	2.5	5	>10	41.26 ^−^	41.26
**T-085 ¤**	1.0	<0.31	<0.31	1.25	0.625	2.5	2.5	5	0.62	1.25	45.42 ^−^	45.42
**T-088 °**	0.5	0.62	<0.31	0.625	<0.31	>10	>10	>10	>10	2.5	36.51 ^+^	73.02
**T-089 °**	0.5	<0.31	0.625	1.25	1.25	>10	>10	>10	0.62	>10	24.27 ^−^	48.54
RMP	<0.06	<0.06	<0.06	<0.06	>2.0	ND	ND	ND	ND	ND	ND	ND

* is methyl ester of quinoxaline 1,4-di-*N*-oxide derivative; ~ is ethyl ester of quinoxaline 1,4-di-*N*-oxide derivative; ¤ is isopropyl ester of quinoxaline 1,4-di-*N*-oxide derivative; ° is n-propyl ester of quinoxaline 1,4-di-*N*-oxide derivative; RMP= rifampicin; IR = isoniazid resistant; ER = ethambutol resistant; SR = streptomycin resistant; RR = rifampicin resistant; ND = not determined; CC_50_ = cytotoxicity on macrophage cell ^−^ at 6 h, ^+^ at 96 h; SI = selectivity index.
